# Joint problem-solving orientation, mutual value recognition, and performance in fluid teamwork environments

**DOI:** 10.3389/fpsyg.2024.1288904

**Published:** 2024-02-13

**Authors:** Michaela Kerrissey, Zhanna Novikov

**Affiliations:** ^1^Harvard TH Chan School of Public Health, Harvard University, Cambridge, MA, United States; ^2^School of Public Health, University of Texas Health Science Center at Houston, Houston, TX, United States

**Keywords:** teamwork, fluid teamwork, healthcare, joint problem-solving, survey

## Abstract

**Introduction:**

Joint problem-solving orientation (JPS) has been identified as a factor that promotes performance in fluid teamwork, but research on this factor remains nascent. This study pushes the frontier of understanding about JPS in fluid teamwork environments by applying the concept to within-organization work and exploring its relationships with performance, mutual value recognition (MVR), and expertise variety (EV).

**Methods:**

This is a longitudinal, survey-based field study within a large United States healthcare organization *n* = 26,319 (2019 response rate = 87%, 2021 response rate = 80%). The analytic sample represents 1,608 departmental units in both years (e.g., intensive care units and emergency departments). We focus on departmental units in distinct locations as the units within which fluid teamwork occurs in the hospital system setting. Within these units, we measure JPS in 2019 and MVR in 2021, and we capture EV by unit using a count of the number of disciplines present. For a performance measure, we draw on the industry-used measurement of perceived care quality and safety. We conduct moderated mediation analysis testing (1) the main effect of JPS on performance, (2) mediation through MVR, and (3) EV as a moderator.

**Results:**

Our results affirm a moderated mediation model wherein JPS enhances performance, both directly and through MVR; EV serves as a moderator in the JPS-MVR relationship. JPS positively influences MVR, irrespective of whether EV is high or low. When JPS is lower, greater EV is associated with lower MVR, whereas amid high JPS, greater EV is associated with higher MVR, as compared to lower EV.

**Discussion:**

Our findings lend further evidence to the value of JPS in fluid teamwork environments for enabling performance, and we document for the first time its relevance for within-organization work. Our results suggest that one vital pathway for JPS to improve performance is through enhancing recognition of the value that others offer, especially in environments where expertise variety is high.

## Introduction

In today’s specialized and fast-paced world, organizations increasingly rely on fluid teamwork. Individuals often come together quickly and change frequently based on the needs of the organization or the nature of the task at hand (Bushe and Chu, 2011; Li and van Knippenberg, 2021). This is common in industries from engineering to healthcare, where networks of diverse experts must be drawn upon to accomplish complex work in the moment (Burke et al., 2004; Edmondson and Nembhard, 2009; Retelny et al., 2014). Teamwork in these settings can offer advantages of expertise pooling, knowledge integration, and shared accountability (Cummings, 2004; Dahlin et al., 2005). Doing so fluidly may enable more efficient and adaptive use of expertise than stable team membership because individuals with distinct expertise can rapidly come and go as the need for their input arises or dissipates. This helps to address issues like urgency (Klein et al., 2006), complexity (Huckman et al., 2009), schedule shifts (Valentine and Edmondson, 2015), and surprises (Bechky and Okhuysen, 2011). However, fluid teamwork environments present challenges for organizational leaders who must establish conditions that enable effective teamwork, calling for new research to identify and understand the factors that are supportive (Salas et al., 2018; Kerrissey et al., 2020).

Teams are groups of individuals who interact to pursue a common goal (Salas et al., 2008a). Richard Hackman described “real” teams as teams that have stable and bounded membership, such that it is clear who is on the team and membership does not shift dramatically over time (Hackman, 2002). Research has since identified stable teams as being advantageous for performance, suggesting that stable teams’ members gain a familiarity over time that confers a better understanding of one another’s strengths, weaknesses, backgrounds, and habits, which can stimulate both cognitive and social benefits (Muskat et al., 2022). For this reason, it is often exhorted that teams be designed to remain relatively stable in order to derive the benefits of familiarity for performance.

However, scholars over the past decade have noted that many dynamic work settings make stable and fixed team membership hard to achieve (Edmondson, 2012; Tannenbaum et al., 2012; Mortensen and Haas, 2018; Li and van Knippenberg, 2021). Fluidity has been described as the presence of shifting team members, i.e., individuals moving on or off the team, though the term at times is also used to refer to *ad hoc* or short-duration teams (Huckman and Staats, 2011; Dibble and Gibson, 2018). For clarity, we use fluidity to refer to membership change and “short duration” to refer to brief team lifespans (though note that, in real world settings, fluidity and short duration often overlap considerably). At the extreme of fluidity in teamwork, individuals may team up together in pursuit of shared goals on the fly or with such a short duration or high degree of individual turnover that ongoing familiarity as a coherent unit becomes elusive—what has been called teaming (Edmondson, 2012) or dynamic participation (Mortensen and Haas, 2018).

Fluid teamwork can offer advantages in adaptiveness and efficiency because individuals are able to come and go as their contributions to a task or goal are needed, but it can also undermine familiarity and its potential benefits to teamwork. For example, it can limit the development of shared mental models and cohesion, which ordinarily help individuals to see joint work similarly and help them to depend upon and reciprocate with one another (Bushe and Chu, 2011). The challenges of fluid teamwork are especially notable in the presence of varied expertise, as familiarity is important for bridging the knowledge differences that separate experts (Kerrissey et al., 2021). In such circumstances, behaviors and orientations to collaboration may be especially valuable because they set expectations about whether and how to team up with others to share information, coordinate, and pursue overlapping goals, even when the structural conditions for ongoing, stable teamwork are not present (Edmondson and Harvey, 2017). Amid calls for research on fluid teamwork for years (Cronin et al., 2011; Wageman et al., 2012; Dibble and Gibson, 2018), there is a particular need for research that explores the contexts in which highly fluid teamwork transpires and that identifies factors that aid performance in the face of considerable barriers.

In this study, we explore the unit conditions that may enable fluid teamwork to thrive, focusing on units within a context known for highly fluid teamwork: health care delivery. Many have written about the challenges of fluid teamwork in health care (Bushe and Chu, 2011), such as shifting task needs due to the emergent nature of many health conditions, the presence of multi-disciplinarity (and its increase with expansion of medical expertise and the addition of new allied health roles), patient-centered frameworks that build unique clinician and staff teams around each patient’s needs, and increasing policy emphasis on team-based care (Andreatta, 2010; Bedwell et al., 2012; Kerrissey et al., 2023). We focus on hospital-based care across units, such as emergency departments, medical intensive care units, surgical intensive care units, and transplant units, because of the common occurrence of shifting sets of individuals teaming up in service of a specific patient during their stay at the hospital. For instance, one study found that the average patient sees 17.8 professionals during a hospitalization (with a range of 5–44), and these individuals come and go throughout the stay as needed and available (Whitt et al., 2007).

Past research in such settings has detailed how fluid teamwork manifests. For example, teamwork shifts around patients in the emergency department with rotating work schedules as nurses and attending physicians clock in and out or specific consulting expertise is brought in for a unique need (Valentine and Edmondson, 2015). Other research has described how intensive care units rely on shifting teamwork across core and peripheral members who may experience brief synchronous periods of work (Mayo, 2022). Aligning with the perspective that health care teamwork often entails fluidity, we sought to examine organizational units (departments in distinct locations) to explore factors that managers and leaders may find useful in promoting effective teamwork in fluid settings and that would be measurable across a large number of work units in future research.

Specifically, we focus on joint problem-solving orientations (JPS)—defined as emphasizing problems as shared and viewing solutions as requiring co-production—as a factor that has been found to promote performance in fluid teamwork settings and for which empirical and theoretical development remains nascent (Kerrissey et al., 2021). Connecting with traditional research that has illuminated the value of shared orientations in more stable teams (Driskell and Salas, 1992; Eby and Dobbins, 1997), the concept of JPS is especially relevant for fluid teamwork because it captures both the perceived jointness of the problem faced and the willingness to resolve it together, even without the luxury of stable team membership or fully aligned goals. Initial research on JPS was conducted in unique fluid teamwork settings—cross-sector, cross-organizational teams, and, later, in a computer simulation about a shopping task (Kerrissey et al., 2021). Other research on fluid teams has been conducted in unique contexts, such as crowdsourced software coding (Retelny et al., 2014). However, much fluid teamwork is more mundane, occurring within the bounds of organizations day-to-day, from software development (Huckman et al., 2009) to health care delivery (Bedwell et al., 2012). In these settings, establishing mechanisms and conditions that enable fluid teamwork to yield performance is vital for overcoming challenges and improving performance. This is especially important for work that relies on experts, who are known to face challenges in moving beyond their individual expertise to fully collaborate (Reyes and Salas, 2019). However, we know little about both the mechanisms through which JPS affects performance and the boundary conditions that shape its effectiveness within organizational units where fluid teamwork is common.

Drawing on multi-year field data from over 1,600 organizational units within a large, geographically-distributed healthcare organization, this paper makes two primary extensions. First, we apply JPS within work units in which fluid teamwork is common, examining its shared presence within organizational units and exploring its relationship with performance in this context. This perspective aligns with the conceptual claim that organizational environments affect teamwork (Salas et al., 2018), alongside the practical reality that departments in hospitals are cogent entities that are used internally to structure work. This makes measurement of JPS within and across departments plausibly informative. Second, we test hypotheses about how JPS affects performance, proposing mutual value recognition (MVR) as a mediator and expertise variety (EV) as a moderator. We focus on MVR as describing the extent to which people recognize (i.e., respect, trust, and listen to) the value that others bring to collaboration. This may be vital for producing performance in fluid teamwork environments where diverse experts draw on distinct languages and norms (Hall, 2005) and may face differences in near-term goals and commitments (Bushe and Chu, 2011). In facilitating a shared focus on solving joint problems, JPS may allow individual experts to better and more rapidly recognize the value that others offer. Our specific hypotheses are detailed in the sections that follow.

## Hypotheses

**Figure 1:**
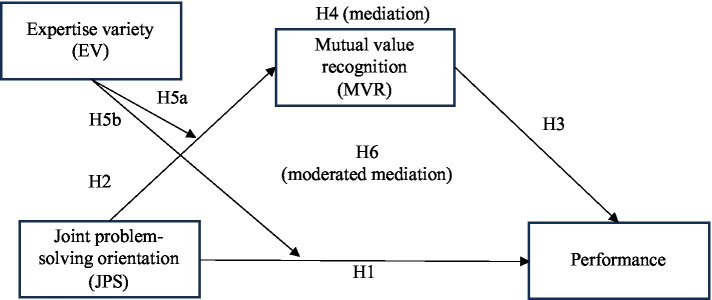
Hypothesized research model.

We build out a set of hypotheses to propose a moderated mediation model (Figure 1), beginning with a main effect of JPS on performance, followed by a set of hypotheses pertaining to MVR as a mediator of that relationship. We conclude with moderation by expertise variety, hypothesizing that more variety heightens the positive relationship between JPS and both MVR and performance.

### Joint problem-solving orientation

Past research has found that joint problem-solving orientation (JPS) is associated with improved work quality in fluid cross-boundary teams (Kerrissey et al., 2021). This has two interrelated aspects: problem-solving and jointness. For problem-solving, seeking help with problem-solving tasks is central to knowledge-intensive work because it enables employees to address and complete complex tasks, thereby directly enhancing performance (Hargadon and Bechky, 2006). The focus not only on problems but also on solving them further emphasizes the value of capturing the willingness and tendency for collaborators to move beyond venting (Rosen et al., 2021) and toward solutions.

The aspect of jointness, though related, is distinct, as individuals may seek help and advice for problem-solving, but that does not guarantee that they do so in a way that implies a shared sense of problem ownership among the asker and receiver of problem-solving assistance. The jointness aspect of JPS refers to this shared emphasis and understanding that problems are mutually faced and require solving together. Jointness is important because of a tendency toward separation among loosely affiliated people; for example, social categorization theory suggests that individuals tend to view others with shared goals, motivations, and priorities as the ingroup and to categorize those who do not as the outgroup (Harrison and Klein, 2007). In related literature, establishment of collective orientation among team members, even in stable teams, has been identified as an important factor in team effectiveness (Driskell and Salas, 1992; Eby and Dobbins, 1997; Hagemann and Kluge, 2017). For instance, research on computer-based simulations of complex teamwork tasks (e.g., extinguishing forest fires and protecting houses) found that, among a set of variables including trust and cohesion, only joint orientation of team members positively affected team performance (Hagemann and Kluge, 2017). Other research on creative teams has found that teams that overcome asymmetries in psychological ownership of their ideas to generate collective ownership have more early successes (Gray et al., 2020).

In line with Kerrissey et al. (2021), we posit that a joint orientation toward problem-solving is particularly relevant for fluid, knowledge-intensive teamwork contexts when diverse experts come together rapidly to solve problems. Kerrissey and co-authors examined this phenomenon in the extreme context of cross-sector, cross-organization teams that form *ad hoc* to solve pressing societal problems. We adapt their logic here to hypothesize that the relationship holds even in more ordinary work contexts (i.e., organizational units in health care). Our first hypothesis thus seeks to replicate the finding that JPS is positively associated with performance, but in the context of organizational work units where fluid teamwork occurs.

H1: JPS is positively associated with performance.

### Mutual value recognition as a mediator

Amid fluid teamwork, the need to swiftly establish a common understanding of what others offer becomes paramount (Bushe and Chu, 2011), especially in the presence of expertise differences (Reyes and Salas, 2019). Beyond a direct effect of JPS on performance through the concrete solving of organizational problems that would otherwise directly hinder performance, we hypothesize that the relationship of JPS to performance is also mediated through a greater recognition of the value that others in their environment offer. Extensive research shows that different expertise areas bring different values, perspectives and technical languages (Carlile, 2004), including in health care (Hall, 2005). Gaining familiarity with one another by working together over time can improve performance (Huckman and Staats, 2011). However, in fluid expertise-driven work contexts where individuals fill roles in shifting sets based on their training (e.g., a nurse acting as a nurse across several teams, and being replaced by other nurses as needed), we posit that JPS enables people to better recognize the value in what other roles and expertise areas have to offer. In spurring problem and solution-focused collaborative work through shared recognition of problems as joint, JPS may help highly trained experts gain real-world experience with and respect for others’ work contributions.

H2: JPS is positively associated with MVR.

H3: MVR relates positively to performance.

H4: MVR mediates the relationship between JPS and performance.

### EV as a moderator

Expertise variety (EV) refers to heterogeneity among members of an interdependent work group who have each accumulated domain specific-knowledge, encompassing variations in functional role or educational background and skill (Ericsson and Smith, 1991). On the one hand, the presence of varied expertise offers the advantage of a more heterogeneous pool of task-relevant perspectives and informational resources to draw from, which serves to enhance team performance (Van Knippenberg et al., 2004). On the other hand, the presence of differing training or functional backgrounds can create communication and cooperation barriers and heighten relational conflicts, damaging interpersonal relationships and negatively affecting performance (Cronin and Weingart, 2007).

We hypothesize that EV in departments moderates the relationship of JPS with both MVR and performance. When there is high expertise variety within a department, we posit that the effect of JPS on performance and MVR is strengthened, as JPS can enable diverse experts to come together and mutually solve problems despite their differing backgrounds. When there is lower EV, we expect that JPS is still positively related to performance but less essentially so, as individuals with similar backgrounds may not need to rely on and value others to address problems collaboratively. Similarly, the benefit of JPS for performance that flows through MVR is likely especially important amid EV because the more experts present the more important it is likely to be that individuals value what others offer.

H5: JPS in the presence of greater EV is related to greater MVR (H5a) and greater performance (H5b).

H6: There is a moderated mediation that explains the relationship between JPS and performance, with MVR mediating the JPS-performance relationship and EV moderating the JPS-MVR and JPS-performance relationships.

## Methods

### Context

We collected data from a large, United States-based organization with over 20 hospitals, over 200 outpatient locations, and over 13 million patient encounters in 2022. It is commonly accepted that teamwork is central to most care delivery environments (Rosenbaum, 2019) and that it is typically fluid (Bedwell et al., 2012), in part because healthcare teams often engage varied expertise in response to patient needs (Rosen et al., 2018). This makes a hospital-based healthcare organization an ideal setting for this study.

### Sample and administration

The organizational survey was sent to 45,471 staff. We excluded individuals from our study who were in purely administrative departments to retain a focus on teamwork in patient-serving care, resulting in *n* = 26,319. The sample was composed of an array of expertise areas including patient-facing caregivers and their managers within the organization, which includes senior management, middle management, physicians, nurse practitioners, registered nurses, licensed practice nurses, nursing assistants, and other clinical professionals such as speech, physical and occupational therapists, alongside some security, service and clerical personnel supporting patient-facing departments. The survey was administered to staff electronically in English at two time points (May 2019 response rate = 87%; May 2021 response rate = 80%). The staff respondents were attributed to 1,608 departmental units, which were defined as being within the same department and physical location (in this organization, a single department can cut across several locations). These departmental units were obtained from human resources files. Table 1 presents the sample characteristics (presented for 2019). The study sample had a predominantly female composition (77.25%) and an age distribution with a large proportion in the 30–49 years age group (48.62%). A slight majority had 1–10 years of tenure (52.38%). There was a range of expertise areas present, with Registered Nurse being the most frequent (23.85%).

**Table 1:** Demographic statistics of the sample (*n* = 26,319).

Characteristic	(%)
Female	77.25
Male	22.54
Prefer not to Answer/Refused	0.22
Age	
8–29 years	16.45
30–49 years	48.62
50–69 years	34.12
70 years and older	0.60
Tenure	
Less than 1 year of service	9.58
1–10 years of service	52.38
11–20 years of service	23.92
More than 20 years of service	14.13
Expertise variety	
Advanced Practice Nurse (Nurse Practitioner)	4.00
Clerical (secretary, accounts clerk, computer/switchboard operator, etc.)	9.55
Clinical Professional (speech/physical/occupational therapist, etc.)	12.60
Licensed Technical (medical lab/radiation therapy technician, etc.)	4.72
Management (e.g., director, manager, and nurse leader)	7.32
Non-clinical Professional (analyst, accountant, communication, etc.)	10.72
Nursing - Other (Licensed Practical Nurse, Nursing Assistant)	7.44
Nursing—Registered nurse	23.85
Physician	6.39
Security, Protective Services, Police	0.34
Senior Management (e.g., executive director, senior director)	0.85
Service (food/nutrition services, environmental services, etc.)	12.00
Skilled Maintenance (carpenter, electrician, general maintenance, etc.)	0.22

### Measures

All measures were assessed using five-point Likert scales and converted to domain means using the mean of the composite items.

#### Joint problem solving

Through iterative input sessions with organizational staff, we modified the joint problem-solving orientation measure developed by Kerrissey et al. (2021) for relevance within a single organization (the original measure was framed to ask about teamwork across two organizations). The adapted measure retained the theoretical emphasis of problems being seen as shared and solutions being seen as requiring co-production, but the language was modified to reflect departments as the referent unit. It included three items: (1) we view addressing problems as a team effort in this department, (2) when a problem arises, we routinely involve whomever is needed to address it, regardless of their unit or role, and (3) we can rely on people in other departments to address problems with us when needed, (*α* = 0.85). JPS was measured in 2019.

#### Mutual value recognition

We measured MVR using relevant items from a validated survey developed for use in care delivery environments to capture affective teamwork across roles, the Primary Care Team Dynamics instrument, which includes 29 items all measured on Likert agreement scales and that are allocated across seven conceptual domains, including conditions for team effectiveness, shared understanding, accountability processes, communication processes, acting and feeling like a team, and perceived team effectiveness (Song et al., 2015, 2017). For the purpose of our hypothesizing in this study, we focused on the teamwork items used to capture valuing, trusting, and respecting others in expertise-diverse healthcare environments, which in the instrument’s measurement scheme fell under the broader theme of “acting and feeling like a team” (this theme also included two other aspects, one pertaining to using team skills and another on communicating information, which were not related to our hypothesizing and thus not measured in this study). In line with our interest in this study on mutually recognizing the value that others can offer, we focused on the three items describing aspects of valuing others, namely, respecting other roles and expertise, trusting each other’s work contributions, and listening to each other. MVR is thus distinct from the adjacent concept of transactive memory systems (TMS), which describes the shared division of cognitive labor in encoding, storage, retrieval, and communication of information (Hollingshead, 2001). MVR focuses not on the cognitive representation and assignment of information from different domains but rather on the recognition that the information from other domains is valuable (respectable, trustable, and worth listening to).

To measure this concept, we used the following items from Song et al. (2015): (1) “People in this department show respect for each other’s roles and expertise,” (2) “People in this department trust each other’s work and contributions,” and (3) “Most of the time people in this department listen to the information that I communicate to them,” (*α* = 0.86). We made slight updates to the original items to reflect the department as the referent entity rather than “team.” We used these items as measured in 2021 to mitigate common method bias concerns with their measurement alongside JPS.

#### Outcome measure

We captured performance in the context of healthcare delivery by measuring staff-perceived care quality and safety, leveraging practical measures used widely within the industry to inform operations and managerial decision-making. Specifically, we used measures from a survey conducted by the health system we studied through a national vendor (Press Ganey), which implements validated employee experience surveys in healthcare. Press Ganey’s industry-oriented research has found that employee perceptions derived from these surveys are related to patient ratings of care as well as hospital financial performance (Buhlman and Lee, 2019). We draw on three measures from their survey, as captured in 2021: (1) “[This organization] provides high-quality care and service,” (2) “[This organization] makes every effort to deliver safe, error-free care to patients,” and (3) “I would recommend [this organization] to family and friends who need care.” These items are conceptually related as markers of performance in healthcare (i.e., that care is both high quality and safe, alongside the general measure of perceived performance based on likelihood of recommending their services to others); they were also empirically related with a high Cronbach’s alpha (*α* = 0.92). For parsimony in presenting our results, we thus operationalize performance as a mean across the three interrelated items; sensitivity analyses examining each item separately yielded similar results.

#### Aggregation of constructs

Because we are interested in the organizational conditions in which fluid teamwork transpires, our measurement is within the department as the local environment that exists within physically located departments, with common management and workers who team up in shifting but overlapping configurations day after day. This approach has been used in prior research on psychological constructs, such as in the study of team climates using psychological safety, which has often been conducted at the departmental level in healthcare (e.g., see Nembhard and Edmondson, 2006).

To justify this aggregation, we calculated within-team agreement parameters and intraclass correlations, and performed a one-way ANOVA for JPS, MVR, and team performance. All scales exhibited significant between-group variance (*F* = 2.36, *p* < 0.01, *F* = 2.61; *p* < 0.01; and *F* = 3.28, *p* < 0.01, respectively). Intraclass correlations were: ICC 1 = 0.10 and ICC 2 = 0.57 for JPS; ICC 1 = 0.11 and ICC 2 = 0.62 for MVR; and ICC 1 = 0.11 and ICC 2 = 0.62 for performance. All scales showed moderate levels of agreement (*rwg* = 0.70 for JPS; *rwg* = 0.71 for MVR; and *rwg* = 0.82 for performance). The ICC and rwg values were consistent with those in team research and considered acceptable for justifying aggregation (Chen and Bliese, 2002; LeBreton and Senter, 2008). The typical values for ICC (1) are 0.01–0.45 and for ICC (2) are 0.45–0.90; values of *rwg* of 0.51–0.70 show moderate agreement, values of 0.71–0.90 show strong agreement, and 0.91–1.00 show very strong agreement (LeBreton and Senter, 2008).

#### Expertise variety

Expertise variety was assessed as a sum of all professional/disciplinary title types in the department [job titles were provided through human resources records and were presented in a consistent fashion such that similar expertise and functional roles were labeled in the same way (e.g., Licensed Practice Nurse, Physician, etc.)]. This variable captures the variety of expertise and reflects the range of specialized knowledge and skills represented among individuals in each department.

### Control variable

As a control measure, we included department size in our analysis, recognizing its established association with performance (Salas et al., 2008b). This was calculated as a sum of all individual people attached to a department-location in the human resources record.

### Analytic procedure

We conducted CFA using structural equation modeling in Stata 15.1 for the individuals answering each item for JPS and MVR (*N* = 24,563), examining root mean squared error of approximation (RMSEA), the chi-squared for the model vs. saturated, the Akaike’s information criterion (AIC), the comparative fit index (CFI), the Tucker Lewis Index (TLI), and the standardized root mean squared residuals (SRMR). We reviewed the descriptive means, standard deviations and correlations of the measures (as depicted in Table 2).

**Table 2:** Means, standard deviations, and bivariate correlations for the research variables.

Variable	Mean	*SD*	1	2	3	4
Dept size	16.04	18.48				
JPS	3.97	0.40	−0.04			
MVR	4.11	0.44	−0.10^**^	0.56^**^		
EV	3.52	1.77	0.47^**^	0.02	−0.01	
Performance	4.47	0.32	−0.06^*^	0.40^**^	0.57^**^	0.09^**^

*n* = 1,608. ^*^p < 0.05; ^**^p < 0.01. Dept size is Department Size by staff count, JPS is joint problem-solving orientation, MVR is mutual value recognition, and EV is expertise variety (count of expertise types).

To test our hypotheses, we conducted a set of regression analyses using SPSS version 27, including a baseline regression, a mediation model, and moderated mediation using two models; we present the underlying regressions for these models in a stepwise fashion for clarity, in a series of Estimated Models (EM), which are each labeled within Table 3. EM1 is a baseline model that includes the control variable of department size only. We then used the PROCESS macro for SPSS developed by Hayes (2018) to estimate the remaining models. To test hypotheses 1 through 4 pertaining to direct effects and mediation, we used a mediation model based on Hayes (2018) mediation “Model 4,” drawing on 5,000 random bootstrap samples. EM2 through EM4 in the results (Table 3) build up the mediation model stepwise.

**Table 3:** Analytic results for baseline, mediation and moderated mediation models.

	Baseline	Mediation model^b^			Moderated mediation models^c^		
Variables	EM^a^ 1	EM 2	EM 3	EM 4	EM 5	EM 6	EM 7
	Performance	Performance	MVR	Performance	MVR	Performance	Performance
	Est. (*SE*)	Est. (*SE*)	Est. (*SE*)	Est. (*SE*)	Est. (*SE*)	Est. (*SE*)	Est. (*SE*)
Dept. size	−0.00^*^ (0.00)	0.00^*^ (0.00)	0.00^**^ (0.00)	0.00 (0.00)	0.00^**^ (0.00)	0.00^*^ (0.00)	0.00 (0.00)
JPS		0.33^**^ (0.02)	0.62^**^ (0.02)	0.10^**^ (0.02)	0.64^**^ (0.02)	0.11^**^ (0.02)	0.10^**^ (0.02)
MVR				0.37^**^ (0.02)		0.36^**^ (0.02)	0.37^**^ (0.02)
EV					0.01 (0.01)	0.02^**^ (0.00)	
JPS^*^EV					0.05^*^ (0.02)	0.02 (0.01)	
*R^2^*	0.00	0.16	0.32	0.34	0.32	0.35	0.34
*F*	5.71^*^	156.89^*^	373.82^**^	269.56^**^	189.80^**^	169.75^**^	269.56^**^

*n* = 1,608. ^*^p < 0.05. ^**^p < 0.01. Dept size is Department Size by staff count, JPS is joint problem-solving orientation, MVR is mutual value recognition, EV is expertise variety (count of expertise types). ^a^EM is estimation model, referring to the analytic model step used to conduct the baseline, mediation, and moderation models. ^b^Results represent the mediation model presented stepwise (Model 4 of Hayes, 2018). ^C^Results represent two moderated mediation models (Models 7 and 8 of Hayes, 2018), which both rely on EM5 for the first part of analysis (Model 7 and Model 8, Hayes, 2018).

For the moderated mediation analysis and testing of Hypotheses 5 and 6, we began with the Hayes mediation “Model 8,” which includes two moderating relationships for the moderator term (one with the mediator and one with the outcome). We used performance as the dependent variable, JPS as the independent variable, MVR as the mediator, and EV as the moderator (presented across EM5 and EM6 in Table 3).

After finding that only one of the hypothesized moderating relationships was statistically significant (between EV and JPS with MVR), we then tested a moderated mediation model that used only that one moderating relationship (excluding the non-significant moderation between EV and JPS with performance) in order to check that the statistically significant moderated mediation holds with one moderating relationship between EV and JPS on MVR (Hayes, 2018; “Model 7”). We present the findings from the moderated mediation with this single moderating relationship across EM5 and EM7. To interpret the form of the interactions in the moderated mediation analysis, we plotted the relationships between JPS, EV and performance/MVR using high and low levels of JPS and expertise at one standard deviation above and below their means (Aiken et al., 1991).

## Findings

### Confirmatory factor analysis

Confirmatory factor analysis yielded a significant model with satisfactory goodness of fit (Hu and Bentler, 1999; *x*^2^ *N* = 24,563, *p* < 0.01, CFI = 0.997, TLI = 0.995, RMSEA = 0.038, SRMR = 0.016, AIC = 302377.974) suggesting that JPS and MVR loaded onto two factors as expected. The two-factor structure yielded a substantially better fit than when JPS and MVR were collapsed into one factor (*N* = 24,563, *p* < 0.01, CFI = 0.701, TLI = 0.501, RMSEA = 0.361, SRMR = 0.182, AIC = 3309). These findings suggest that JPS and MVR are two distinct constructs (Cangur and Ercan, 2015).

### Descriptive statistics

Table 2 summarizes the descriptive statistics and the correlations among the control, independent, and dependent variables at the department level.

### Hypothesis testing

Table 3 presents the results of the baseline, mediation, and moderated mediation models, all of which include department size as a control variable. Consistent with hypothesis 1, we found a significant relationship between JPS and performance (*b* = 0.33, *SE* = 0.02, *p* < 0.01; see EM 2). We also found evidence consistent with hypothesis 2 relating JPS to MVR (*b* = 0.62, *SE* = 0.02, *p* < 0.01; see EM 3). When regressing, JPS and MVR on performance (see EM 4), we found a significant relationship between JPS and performance (*b* = 0.10, *SE* = 0.02, *p* < 0.01), and MVR and performance (*b* = 0.37, *SE* = 0.02, *p* < 0.01). The effects of the mediation pathways are significant as follows: the total effect of JPS on performance = 0.33 (Bootstrapped *SE* = 0.02, with 95% *CI* [0.29, 0.36]), the direct effect of JPS on performance = 0.10 (Bootstrapped *SE* = 0.02, with 95% *CI* [0.06, 0.14]), and the indirect effect of JPS to performance through MVR is = 0.23 (Bootstrapped *SE* = 0.02, with 95% *CI* [0.20, 0.26]). Taken together, these results support Hypothesis 4 pertaining to the presence of mediation.

**Figure 2:**
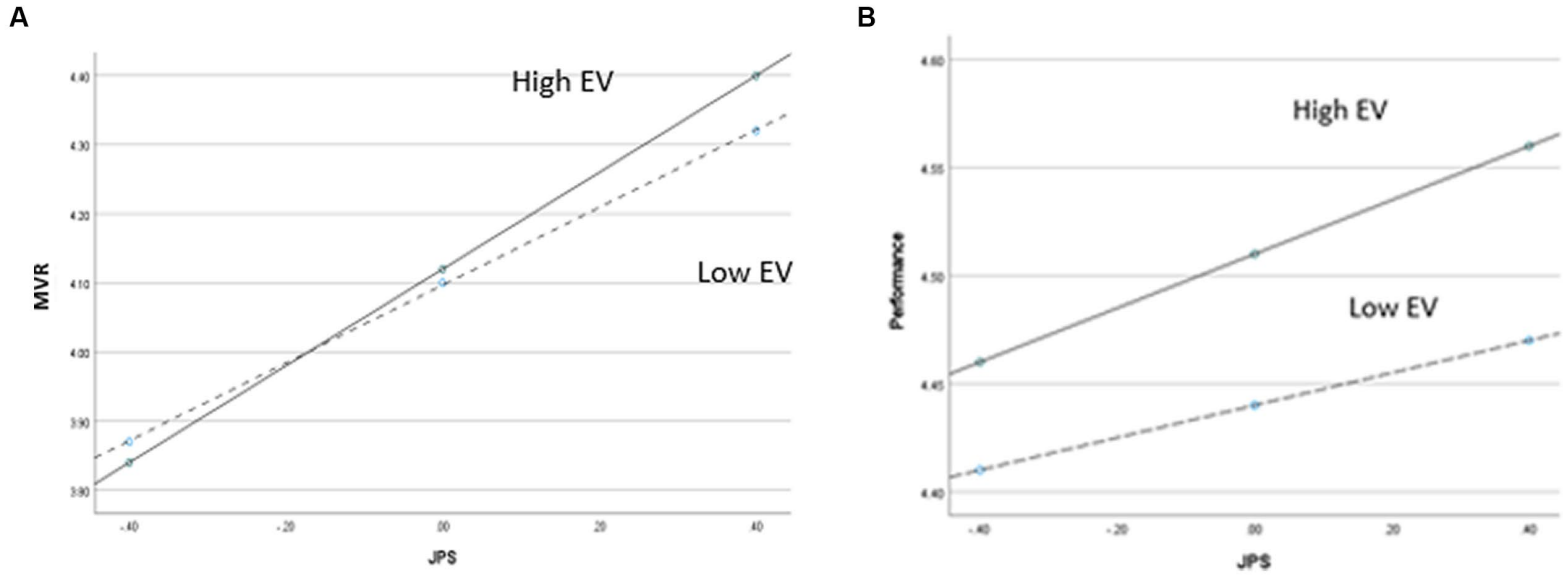
**(A)** Two-way interaction between JPS and EV on MVR as the dependent variable. **(B)** Two-way interaction between JPS and EV on performance as the dependent variable.

For the first part of moderated mediation analysis, we regressed JPS, EV, and the interaction between JPS and EV on MVR (see EM 5). We found a significant interaction (*b* = 0.05, *SE* = 0.02, *p* < 0.05), which supports hypothesis 5a. Figure 2A visually presents the form of the interaction, plotting the relationship between JPS, EV, and MVR using high and low levels of JPS and expertise at one standard deviation above and below their means (Aiken et al., 1991).

For the second part of moderated mediation model, we regressed JPS, MVR, and EV and the interaction between JPS and EV on performance (see EM 6). The interaction between JPS and EV was not statistically significant (*b* = 0.02, *SE* = 0.01, *p* = 0.16). Hypotheses 5b and 6 were thus not supported. Figure 2B visually presents the form of the interaction, plotting the relationship between JPS and EV and performance using high and low levels of JPS and EV at one standard deviation above and below their means (Aiken et al., 1991).

We then re-tested the moderated mediation model while excluding the non-significant moderation between JPS and EV on performance from the model (Model 7, Hayes, 2018). We found a significant relationship between JPS and performance (*b* = 0.10, *SE* = 0.02, *p* < 0.01; Table 2, EM 7), and MVR and performance (*b* = 0.37, *SE* = 0.02, *p* < 0.01). The index of moderated mediation (PROCESS, Model 7; Hayes, 2018) support a moderated mediation model (indirect effect = 0.02, Boot *SE* = 0.01, with 95% *CI* [0.01, 0.03]). The results support a moderated-mediation model with EV moderating the relationship between JPS and MVR, and MVR mediating the relationship between JPS and performance.

## Discussion

This study sought to extend understanding of JPS within an organizational context characterized by fluid teamwork. We found evidence in support of a moderated mediation model, in which JPS was associated with performance directly and through MVR as a mediator, and in which JPS was most strongly related to MVR when expertise variety was high. These findings advance the nascent theory and research on JPS in fluid teamwork environments. They highlight JPS as valuable for organizations seeking to improve performance.

Building upon the recently identified concept of JPS in research on fluid cross-sector teams (Kerrissey et al., 2021), we found that JPS was also associated with performance within organizational work units that rely upon highly fluid teams of experts to conduct complex work. Our results show that this relationship held when controlling for departmental size, and the results indicate that a substantial proportion of the variance was explained even in the parsimonious models that we used (i.e., observing the r-squared terms ranging from 0.34 to 0.35). As an orientation, JPS is focused on the presence of a shared emphasis, focusing on the interpersonal rather than informational aspects of fluid teamwork—particularly, how people approach one another in reference to the work they are doing together and the problems they face. Though connected conceptually and likely empirically, it is thus distinct from other measures that focus on factors like transactive memory systems and information sharing (e.g., Hollingshead, 2001; Mesmer-Magnus and DeChurch, 2009). Our results lend evidence to JPS as a factor worth examining.

Through mediation analysis, we found that part of the relationship between JPS and performance occurred through an enhanced recognition of the value that others can offer in collaborative work. This aligns with the perspective that experts must be able to swiftly establish a common understanding (Reyes and Salas, 2019)—and our findings lend evidence to the idea that JPS may help experts to do this more readily as they team up day-to-day with others. Put practically in an example, this represents the notion that a physician may not necessarily only need to learn afresh what a nurse “knows” but also to recognize that what a nurse knows about a patient from serving at their bedside is an important and valid input to the care process that is worth deliberately incorporating. This type of recognition may often come through familiarity in stable teams; it appears that JPS may also enable it, even without the luxury of stable teamwork over time.

Our findings lend support to EV as a moderator. However, it was only statistically significant for the relationship between JPS and MVR and not for the direct relationship between JPS and performance. The pattern for the moderating relationship between JPS and MVR was notable. As Figure 2A depicts, we found that when JPS was low, high EV resulted in less MVR. As JPS increased, MVR increased for all levels of EV. When JPS was high, organizational units with more EV showed higher MVR than units with lower EV. This suggests that in units with less EV, a little JPS may go a long way to foster MVR, but when there is substantial EV, a relatively high amount of JPS may be needed to expand MVR. This underscores the importance of JPS in highly expertise-varied environments for rapidly establishing awareness of what other expertise domains can contribute. This is especially notable in contrast to the moderation of the direct relationship between JPS and performance, which though not statistically significant implied the potential of a notably different pattern, in which greater EV always strengthened the relationship between JPS and performance, regardless of the level of JPS (Figure 2B). This contrast seems plausible, as having more expertise to draw from extends the pool of task-relevant perspectives and informational resources to draw from, which serves to enhance team performance directly (Van Knippenberg et al., 2004). Our findings suggest that for MVR this advantage is likely to differ, requiring substantial JPS to engender MVR when EV is high.

### Implications for theory and future research

Our findings contribute to the emerging literature on fluid teamwork, for which there have been calls for more research (Tannenbaum et al., 2012; Wageman et al., 2012; Mortensen and Haas, 2018). In exploring JPS within organizational units where highly fluid teamwork is dominant, we found that a factor that was initially studied in the unique context of cross-sector teams remained relevant, and our analyses enhanced our understanding of how it yields performance through the moderated mediation model we test. We nonetheless view our study with an exploratory lens, given the nascency of research on fluid teamwork and the empirical difficulty of studying fluid teamwork at scale within organizations, which often leads to “glimpses” rather than comprehensive pictures of this dynamic phenomenon (Kerrissey et al., 2020). There is a great deal more to explore and learn.

A main contribution of this research is to extend JPS to the organizational work unit context and to better understand how JPS operates and the boundary conditions that might shape its effectiveness in organizational contexts. While we find support for our conceptual model of moderated mediation, there are likely other important boundary conditions and mechanisms that can be proposed and explored in future research. For example, future research might examine how hierarchy and team climate measures such as psychological safety might relate to JPS and performance (Nembhard and Edmondson, 2006). Research is also needed to identify ways to prompt JPS and to do so in a way that further facilitates MVR. One promising avenue may be through interventions focused on reflection; research on interprofessional collaboration, for instance, has underscored the value of reflection in helping individuals loosen the dominance of their tacitly acquired professional identities that prevent them from collaborating more effectively (Wackerhausen, 2009).

While we examine JPS and MVR across a 2-year timeframe, there is great opportunity to examine these relationships with more longitudinal time points and a greater focus on temporal developments. Both theory on teams and theory on problem solving present development as a temporal process; for example, the model of problem solving of Mac Duffie (1997) articulates problem definition, analysis, generation and selection of solutions, testing and evaluation of solutions, and routinization. Future theoretical work might integrate the temporal models around team development and problem-solving fruitfully to conceptualize how JPS unfolds. Further, there is opportunity for in-depth ethnographic research to examine JPS in real-world settings to identify its antecedents. This kind of in-depth longitudinal work may be particularly valuable given the likelihood of mutually reinforcing relationships; although our measurement timeframe and theory suggest that JPS generates MVR, it is also plausible that MVR further reinforces JPS. Indeed, the decision to ask for problem-solving assistance is enhanced by a cognitively-based appraisal of that person (Nebus, 2006). Studies to investigate how team processes dynamically unfold are needed, for instance, event and time-based behavioral observation (Kolbe and Boos, 2019).

Because our purpose in this paper was to explore and extend the concept of JPS for fluid teamwork environments, we focused on JPS alongside a mediator and moderator rather than comparing JPS to alternative factors. We thus do not attempt to make a comprehensive model of team fluidity and performance—there are other factors that matter, and future research can explore how they compare to or interact with JPS. For example, our hypothesizing and findings in support of MVR as a key mediator differ from common explanations in more stable teamwork environments, where for instance collective psychological ownership is thought to be valuable for prompting effort, commitment and sacrifice among members, rather than elements of mutual value recognition (Pierce and Jussila, 2011; Gray et al., 2020). It may be that in highly fluid teamwork among experts commitment mechanisms, though likely present to some degree, are less central because high fluidity may make commitment to any particular team entity less essential. This would be interesting to test in future research. A second, related area of extension could examine positive affective relationships, which are often cited as important factors in intact teams. However, research on problem-solving work has found a performance benefit to seeking out problem solving assistance from “dissonant ties,” i.e., difficult colleagues with whom a relationship may be fraught (Brennecke, 2020). This points to potentially interesting and important differences in the role of positive affective bonds, relative to MVR, in highly fluid teamwork among experts; for instance, it is possible that MVR can develop effectively among dissonant ties, helping to value contributions even when other bonds remain suboptimal. Future research could compare and test these ideas.

### Implications for practice

For practice, our findings suggest that organizational leaders and managers might look to joint problem-solving orientations as a key factor to promote performance within their organizational units where fluid teamwork occurs. This is important given that fluid teamwork is a reality in many highly dynamic, expertise-driven work settings (Mortensen and Haas, 2018). Our research suggests that when fluid teamwork prevents people from gaining in-depth familiarity with other individuals—a key to performance in stable teams (Hackman, 2002)—they may nonetheless through joint problem-solving orientations come to better recognize the value of others’ contributions and thereby generate performance.

For organizations, looking for ways to hire for, foster, measure and reward JPS may be highly valuable. Our measurement of JPS at the departmental level in this study suggests that organizations may use this level of measurement to inform and improve their fluid teamwork in practice, as they may find it onerous or infeasible to track such measures at the team level amid such high fluidity (e.g., in healthcare, it might otherwise require surveying staff for each of the many teams they interact with per day). Moreover, that a department-level measure of JPS has predictive power for performance in a fluid teamwork environment may also offer to practitioners a pragmatic entity for intervention. Consider the alternative for a highly dynamic organizational environment: even if an organization were able to collect data on each fluid team that formed, if those teams are so fluid, distinct and often short-lived as they are in healthcare, then it would nonetheless not be clear who would be responsible for intervening, when, or with whom. That organization might then have beautiful data on which teams perform best, and which need help, only to realize that none of the teams still exist. For this reason, a departmental or similar unit-based measurement approach may be advantageous to intervention within organizations.

### Limitations and future research

This study has limitations. First, while the analysis was conducted across a large number of work units (i.e., departments), it was conducted within one overarching healthcare delivery organization. Future efforts to further test these measures and relationships in other organizations and industries where fluid teamwork is central are needed to inform the generalizability of our findings. There are aspects of healthcare delivery, such as the overarching shared mission of delivering high quality patient care across disciplines, that may make JPS more salient for performance and MVR more relevant as a mediator in this context than others. Second, there are limitations in measuring these concepts at a departmental level. We did not have access to measures of variation by degree of fluidity in teamwork, and presumably there is some variation in degree of fluidity across departments in our study that would be fruitful to explore. Moreover, we recognize that measuring constructs at the departmental unit is imperfect in healthcare, especially as additional teamwork occurs across departments. However, because the preponderance of work occurs within departments (i.e., within an intensive care unit) and because JPS influences the interactions among members teaming up within the department, we believe it is a useful and reasonable simplification. In addition, the consistency and agreement measures (e.g., ICCs and RWGs) we analyzed provided empirical evidence that the key constructs were similar within and different across the departments. Third, future research can further develop the concept and refine the measurement of MVR, given its significant relationship to JPS and performance in our exploratory analysis. Fourth, because we measured JPS and MVR within the department rather than measuring these factors in each fluid team that occurred, our results address the department environment rather than the fluid team as the unit of analysis. While this offers advantages for pragmatism and practice, it is also a limitation for understanding team-level orientations and processes. Future research could fruitfully extend theory in this area by observing JPS as it forms in the moment within fluid teams.

## Data availability statement

The datasets presented in this article are not readily available because this is organizational data with sensitive employee information that authors have access to under condition of not sharing it. Requests to access the datasets should be directed to mkerissey@hsph.harvard.edu.

## Ethics statement

The study involving humans was approved by Harvard University Institutional Review Board. The studies were conducted in accordance with the local legislation and institutional requirements. Written informed consent for participation was not required from the participants or the participants’ legal guardians/next of kin in accordance with the national legislation and institutional requirements.

## Author contributions

MK: Conceptualization, Data curation, Investigation, Methodology, Project administration, Writing – original draft, Writing – review & editing. ZN: Conceptualization, Data curation, Formal analysis, Methodology, Visualization, Writing – review & editing.
